# A phase I clinical trial of human embryonic stem cell‐derived retinal pigment epithelial cells for early‐stage Stargardt macular degeneration: 5‐years' follow‐up

**DOI:** 10.1111/cpr.13100

**Published:** 2021-08-04

**Authors:** Shi‐Ying Li, Yong Liu, Lei Wang, Fang Wang, Tong‐Tao Zhao, Qi‐You Li, Hai‐Wei Xu, Xiao‐Hong Meng, Jie Hao, Qi Zhou, Liu Wang, Zheng‐Qin Yin

**Affiliations:** ^1^ Southwest Hospital/Southwest Eye Hospital Third Military Medical University (Army Medical University) Chongqing China; ^2^ Key Laboratory of Visual Damage and Regeneration & Restoration of Chongqing Chongqing China; ^3^ State Key Laboratory of Stem Cell and Reproductive Biology, Institute of Zoology Chinese Academy of Sciences Beijing China; ^4^ Stem Cell and Regenerative Medicine Innovation Institute Chinese Academy of Sciences Beijing China; ^5^ National Stem Cell Resource Center Chinese Academy of Sciences Beijing China; ^6^ University of Chinese Academy of Sciences Beijing China

**Keywords:** clinical trial, embryonic stem cell, human, macular degeneration, retinal pigment epithelial cells

## Abstract

**Objectives:**

To evaluate the long‐term biosafety and efficacy of transplantation of human embryonic stem cells‐derived retinal pigment epithelial (hESC‐RPE) cells in early‐stage of Stargardt macular degeneration (STGD1).

**Materials and methods:**

Seven patients participated in this prospective clinical study, where they underwent a single subretinal transplantation of 1 × 10^5^ hESC‐RPE cells in one eye, whereas the fellow eye served as control. These patients were reassessed for a 60‐month follow‐up through systemic and ophthalmic examinations.

**Results:**

None of the patients experienced adverse reactions systemically or locally, except for two who had transiently high intraocular pressure post‐operation. Functional assessments demonstrated that all of the seven operated eyes had transiently increased or stable visual function 1‐4 months after transplantation. At the last follow‐up visit, two of the seven eyes showed visual function loss than the baseline; however, one of them showed a stable visual acuity when compared with the change of fellow eye. Obvious small high reflective foci in the RPE layer were displayed after the transplantation, and maintained until the last visit. Interestingly, three categories of patients who were classified based on autofluorescence, exhibited distinctive patterns of morphological and functional change.

**Conclusions:**

Subretinal transplantation of hESC‐RPE in early‐stage STGD1 is safe and tolerated in the long term. Further investigation is needed for choosing proper subjects according to the multi‐model image and function assessments.

## Graphical abstract

Transplantation of human embryonic stem cells‐derived retinal pigment epithelial (hESC‐RPE) cells in the patients with early stage of STGD1 is safe and tolerated in the long term. Objective visual functions test after transplantation were mostly remained as the preoperative levels or improved transiently in some cases. Morphology test showed pigmentation in the fundus and high reflective foci in the RPE layer by OCT. Moreover, a new fundus autofluorescence classification was applied to group the subjects for stem cell transplantation and monitored the clinical outcomes.

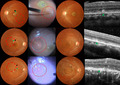

## 1 INTRODUCTION

Stargardt macular degeneration (STGD1), also known as juvenile macular degeneration, is the most common monogenic hereditary macular/retinal dystrophy caused by autosomal recessive mutations in the *ATP binding cassette subfamily A member 4* (*Abca4*) gene in chromosome 1.^1^, ^2^ This gene encodes the Rim protein (RmP or ABCA4), which is specifically expressed in photoreceptor cells and retinal pigment epithelium (RPE) cells. Mutations in the photoreceptor‐specific flippase transporter ABCA4 leads to an accumulation of the toxic *N*‐retinylidene‐*N*‐retinylethanolamine (A2E), resulting in atrophy of the RPE and in the death of photoreceptor cells.^2^, ^3^, ^4^ Despite the high incidence of this disease, no curative treatments for it have been developed. While gene replacement therapies involving adeno‐associated virus (AAV) have been successfully used for some ocular genes, the 6.8 kb cDNA of ABCA4 is too large to be packaged into AAV vectors. Alternatively, lentiviral vectors, non‐viral compacted DNA nanoparticles, CRISPR/Cas9‐mediated genome editing of patient skin cell‐derived‐induced pluripotent stem cells (iPSCs) or human embryonic stem cells (hESCs) could be used in gene‐ or cell‐based therapies.^5^, ^6^, ^7^ Recent studies have demonstrated that both genome‐edited iPSCs and hESCs could be differentiated into retinal pigment epithelial (RPE) cells in vitro for transplantation. Moreover, the RPE cells implanted into human subjects are well‐tolerated and could potentially restore some vision loss in STGD1 patients.^8^, ^9^, ^10^, ^11^ Thus, transplantation of hESC‐RPE cells may become a promising new treatment option for macular degeneration. However, the long‐term safety of stem cell implantation into human eyes had remained unclear, and the field lacked an objective visual function evaluation system for macular degeneration patients.

In our previous studies, we successfully generated RPE cells from clinical grade (CTS)‐hESCs, which met the standard requirements for clinical applications.^12^, ^13^ We demonstrated that the CTS‐hESC‐RPE cells, transplanted into the subretinal space of the Royal College of Surgeons (RCS) rats with inherited retinal degeneration and light‐damaged pigs, protected the animals against retinal degeneration.^14^, ^15^ In a recent clinical trial, we implanted CTS‐hESC‐RPE cells into the subretinal space of patients for the treatment of wet age‐related macular degeneration (wet‐AMD) due to neovascular disruption.^8^ With optimized perioperative management and surgical protocols, wet‐AMD patients who underwent the transplantation showed a new RPE‐like cell layer in a previously damaged retinal area, without adverse health events. Moreover, visual function test results indicated partial vision improvement.^8^ Our studies indicated that RPE cell replacement therapy is safe and feasible for wet‐AMD, and possibly for other macular degeneration diseases.

In this study, we extended our hESC‐RPE cell‐based therapy to patients with early‐stage STGD1 and conducted a phase I clinical trial. We employed a new fundus autofluorescence classification approach to group the subjects after stem cell transplantation, and we monitored their clinical outcomes. The patients were followed up for 5 years to evaluate the long‐term safety and efficacy of the hESC‐RPE therapy.

## 2 MATERIALS AND METHODS

### 2.1 *hESC‐RPE* preparation

The hESCs, specifically the Q‐CTS‐hESC‐2 cell line, were provided by the Institute of Zoology, Chinese Academy of Sciences and were authenticated by the Institute of Pharmaceutical and Biological Products Certification in China. The CTS‐hESCs were differentiated into RPE, and the CTS‐RPE cells were expanded, purified and certified by the GMP Laboratory of Cell Biotherapy Center, Southwest Hospital of Army Medical University, as described previously.^8^ Each vial contains less than 0.5 EU/mL endotoxin and was pathogen‐free (based on the results of the tests for fungi, bacteria, mycoplasma, syphilis, human immunodeficiency virus, and hepatitis B and C). The CTS‐RPE cells had a >95% viability.

### 2.2 Patient recruitment and Study design

Seven patients (two males and five females) who met the inclusion criteria were enrolled in this prospective study, which was an open‐label, self‐control, and single‐centre study conducted from May 2015 to May 2020. Each participant underwent a single subretinal transplantation of 1 × 10^5^ hESC‐RPE cells into the operated eye, and the fellow eye served as control. We chose the eye with worse best‐corrected visual acuity (BCVA). If the BCVA was same in both eyes, the eye with worse retinal electroretinography (ERG) and autofluorescence (AF) was chosen. The patients were followed up for 9‐60 months after implantation. This study was approved by the Medical Ethics Committee of Southwest Hospital, Army Medical University (2015‐18). The clinical trial was registered at the clinicaltrials.gov with a reference number NCT02749734.

### 2.3 Surgical procedure

Surgical vitrectomy and subretinal injection were carried out as reported.^8^, ^16^ In brief, a three‐port pars plana vitrectomy was performed; this procedure involved both the creation of a complete posterior vitreous detachment and total vitreous excision. A small amount of saline was infused using a 41‐gauge injection cannula (Bausch & Lomb Storz) into the sub‐retinal space to detach the temporal retina, which is located next to the macula. A total of 10^5^ Q‐CTS‐hESC‐2‐RPE cells suspended in 100 μL volume were slowly injected into the sub‐retinal space, creating a localized dome‐shaped retinal detachment in the macular area (Video S1). Then the silicon oil was tamponaded after air–fluid exchange. After the surgery, the patient remained in a supine position overnight until the subretinal fluid was absorbed; thereafter, the patient changed to a prone position, which was maintained for 1 week. The silicon oil was removed after 3 months of transplantation. To inhibit potential immune response and inflammation, we administrated immunosuppression drugs to the patients before and after surgery as described previously.^8^, ^17^ Specifically, the patients received oral mycophenolate mofetil (500 mg, bid), tacrolimus (0.1 mg/kg/d, bid) and prednisone (0.5 mg/kg/d, qd) 1 week before surgery. After surgery, mycophenolate mofetil was given for 4 weeks. Tacrolimus was titrated to keep its serum levels within 3‐7 ng/mL and was maintained until the 12th week. The prednisone dosage was decreased (0.25 mg/kg/d, qd) at the 4th week and then stopped at the 12th week.

### 2.4 Clinical evaluation

**FIGURE 1:**
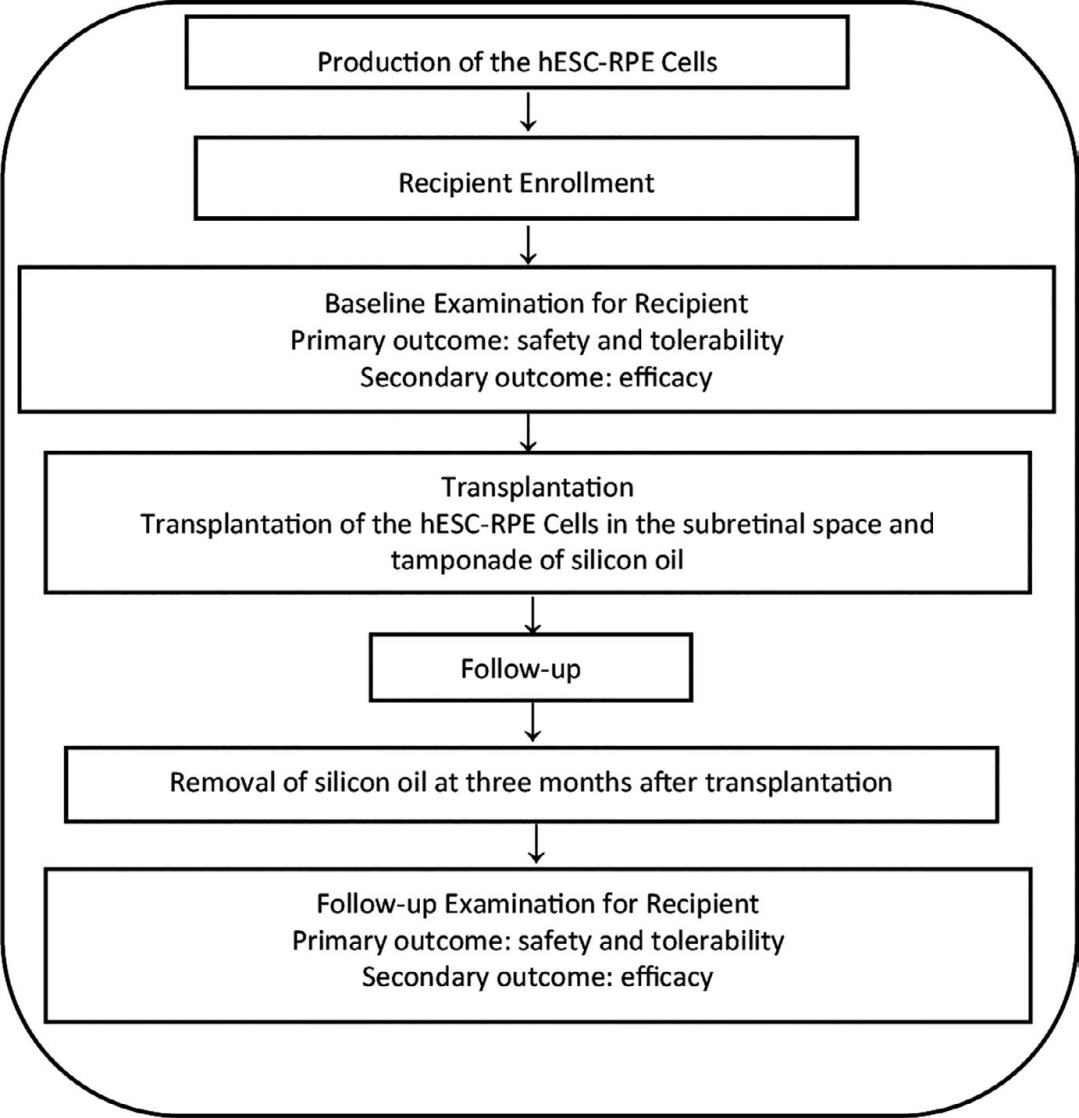
Flow chart of the experimental procedure

The clinical examination protocol is summarized in the Figure 1. The primary outcomes were the safety and tolerability of hESC‐RPE cells in patients with early‐stage Stargardt macular degeneration, which was monitored by systemic and ophthalmic examinations. The systemic examinations included chest X‐ray, electrocardiogram and blood test. The ophthalmic examinations included slit‐lamp biomicroscopy, optical coherence tomography (OCT) in the retina, fundus photography and fundus autofluorescence (AF). The secondary outcome was the efficacy of hESC‐RPE cells, which was monitored by the ophthalmic examinations including best‐corrected visual acuity (BCVA) using the Early Treatment Diabetic Retinopathy Study (ETDRS) alphabet chart, tonometer, full‐field electroretinography (ffERG), mutifocal electroretinography (mfERG), pattern visual evoked potential (PVEP) and visual field examinations. The BCVA is regarded as stable if the EDTRS score differs by less than five letters compared with that of the same eye before operation (baseline) or when the difference of EDTRS score changing between operated eye and fellow eye is less than five letters from the baseline to the visiting time point. All participants were assessed using the Vision‐Related Quality of Life and Visual Function‐25 (VFQ‐25) questionnaire. More detailed clinical evaluation methods are stated in the [Link], [Link], [Link], [Link], [Link].

### 2.5 Statistical analysis

Data were analysed using SPSS17.0 software and were expressed as mean ± standard deviation. Visual acuity, visual field and amplitudes as well as peak time of electrophysiology were measured during the follow‐up visits. The visual parameters for the same eye and which were obtained at different time points (follow‐up visits) were compared with the baseline measurement to evaluate any vision improvement. Moreover, the ETDRS letter scores of the fellow eye during BCVA assessments, which were conducted at the same time points, were compared with the baseline. Hybrid model test was employed in the statistical analyses. *P* < .01 indicated a significant difference between two groups. [Link], [Link], [Link], [Link], [Link] is available at Cell Proliferation's website.

## 3 RESULTS

Seven patients including two males and five females aged 19‐27 years (median age: 23 years) were enrolled in this study. These STGD1 patients underwent surgery, wherein one eye was operated (operated eye) and the other eye served as a control (fellow eye). These patients were followed up for 9‐60 months. As shown in Table 1, all patients were characterized as ffERG type 1 before transplantation.^1^ They had stable vital signs, and they did not show any adverse conditions, such as allergy, immune rejection, fever, headache, or other systemic adverse reactions after subretinal hESC‐RPE injection (Table S3). Moreover, they did not experience any severe local complications, such as endophthalmitis or retinal detachment. Systemic examination revealed that neither tumour‐like appearance nor elevated levels of tumour markers in the bloodstream were detected throughout the treatment period. However, 1‐2 months after operation, two of the seven patients (P4 and P7) had a transiently high intraocular pressure ranging from 26 to 32 mm Hg, which was relieved by eye drops and cured after silicone oil removal 3 months after transplantation. Other adverse events, including conjunctival haemorrhage, hyperaemia and eye sting pain, were transient and did not require any intervention (Table S3). The VFQ‐25 questionnaire did not reveal any significant changes before and after stem cell transplantation.

**TABLE 1:** Patients information

Participant number	Age (years)	Duration	Sex	Genotype pathogenic variants on *ABCA4* gene	ACMG class	Gene typing	Operated eye	Baseline Visual Acuity ETDRS (letters)^a^		ERG group	AF category	Follow‐up time (month)
								Operated eye	Follow eye			
P1	21	5	F	c.5882G > A (p.Gly1961Glu)	P (PS1, PM3, PM5, PP2, PP3, PP5)	B	OS	39	39	Group 1	Category Ⅰ	42 mo
				c.1222C > T (p.Arg408Ter)	P (PVS1, PM2, PP5)							
P2	19	8	F	c.1819 G > A (p.Gly607Arg)	P (PS1, PM2, PM5, PP2, PP3, PP5)	C	OD	40	42	Group 1	Category Ⅲ	60 mo
				c.2261T > C (p.Phe754Ser)	P (PS1, PM2, PM3, PP2, PP3, PP5)							
P3	20	5	F	c.6563T > C (p.Phe2188Ser)	P (PS1, PS4, PM2, PP1, PP2, PP3)	C	OD	40	40	Group 1	Category Ⅱ	60 mo
				c.3749T > C (p.Leu1250Pro)	LP (PM1, PM2, PM3, PP1, PP3)							
P4	22	10	F	c.4948G > T (p.Glu1650Ter)	P (PVS1, PS1, PM2, PP2, PP3)	B	OS	44	44	Group 1	Category Ⅱ	60 mo
				c.3482G > A (p.Arg1161His)	P (PS1, PM1, PM2, PM3, PP2, PP3)							
P5	27	2	M	c.4352 + 1G > A (p)	P (PVS1, PM2, PP3, PP5)	B	OD	20	47	Group 1	Category Ⅲ	12 mo
				c.1748A > C (p.Lys583Thr)	LP (PM1, PM2, PM3, PP2, PP3)							
P6	27	11	M	c.6563T > C (p.Phe2188Ser)	P (PS1, PM1, PM2, PP2, PP3, PP5)	C	OS	44	44	Group 1	Category Ⅰ	42 mo
				c.3476T > C (p.Leu1159Ser)	P (PS1, PM1, PM2, PP2, PP3)							
P7	27	9	F	c.6328T > C (p.Trp2110Arg)	LP (PM1, PM2, PM3, PP2, PP3)	C	OS	23	35	Group 1	Category Ⅲ	9 mo
				c.2894A > G (p.Asn965Ser)	P (PS1, PM1, PM2, PM5, PP2, PP3)							

Abbreviations: ACMG, American College of Medical Genetics; AF, autofluorescence; ERG, electroretinography; LP, likely pathogenic; P, pathogenic.

^a^ Baseline Visual Acuity was recorded in ETDRS (letters).

**FIGURE 2:**
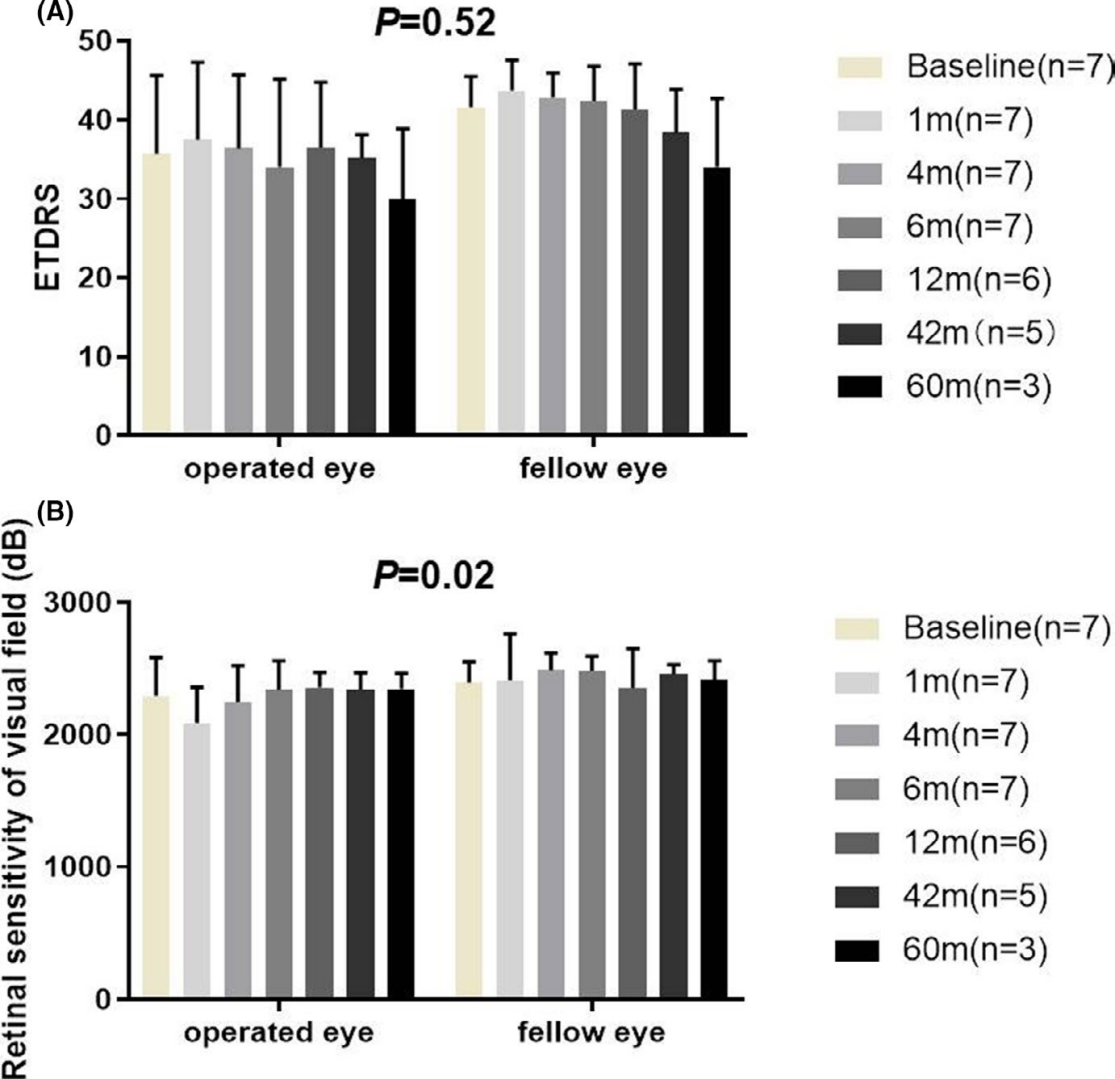
Best‐corrected visual acuity (BCVA) and visual field in patients with Stargardt macular degeneration (STGD1). There was no significant difference in BCVA by ETDRS letters (A) or the overall retinal sensitivity of visual field (B) between operated eye and fellow eye at each time point

To evaluate the retinal function, we tested the patients' visual acuity using the ETDRS, and we found that all of the seven operated eyes remained stable in terms of visual acuity, compared with the fellow eyes at the same time point (*P* = .52, Figure 2). Of the seven patients, two (29%, P3 and P5) regained vision at the 1st month and one patient (14%, P5) regained vision at the 4th month of follow‐up. Three of those seven patients (42%, P2, P3 and P6) had ETDRS letter scores that were lower by more than five letters compared with the baseline during the last visit, indicating visual acuity loss. However, only one of them (14%, P2) was considered to have a significant visual acuity decrease after stem cell transplantation, considering the difference in EDTRS scores between the operated eye and the fellow eye from the baseline to the visiting time point (Table S1). Moreover, there was no significant difference in the overall retinal sensitivity of the visual field of the treated eyes before and after operation, nor between the treated eyes and the fellow eyes (*P* = .09, Figure 2).

**FIGURE 3:**
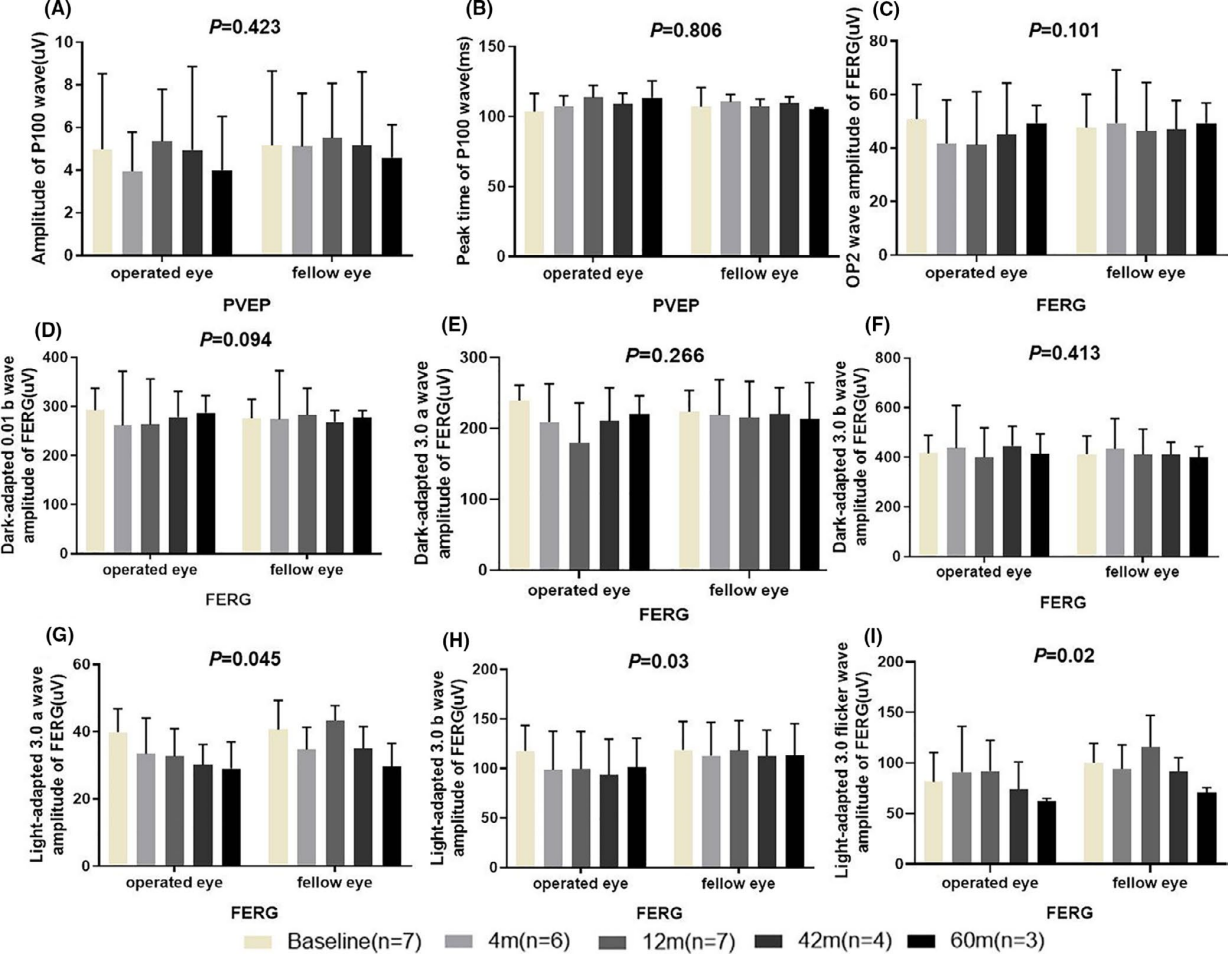
Changes in the visual electrophysiology of the operated and fellow eyes. The amplitude (A) and peak time (B) of P100 wave in the pattern visual evoked potential (PVEP) of the operated and fellow eyes did not significantly differ. Full field electroretinography (ffERG) showed that there was no significant difference between the operated and fellow eyes at same the time point in terms of the amplitudes of Op2 wave (C), b‐wave in dark‐adapted 0.01 (D), a‐wave (E) and b‐wave (F) in dark‐adapted 3.0, a‐wave (G) and b‐wave (H) in light‐adapted 3.0, and wave amplitude of light‐adapted 3.0 flicker (I)

Subsequently, we evaluated the patients' visual acuity by using the PVEP functional test. We found that the patients' amplitude of P100 wave decreased transiently within the first 4 months after transplantation, but it recovered to the baseline level between the 12th and 42nd month of follow‐up. At the last visit (60 month), the P100 wave of PVEP decreased slightly. No significant difference in the amplitude of P100 wave was observed between the operated eyes and the fellow (control) eyes (*P* = .565) (Figure 3A). The peak times for the treated eyes were stable during the follow‐up visits and were comparable with those of the fellow eyes (*P* = .98) (Figure 3B). In the ffERG test results, no significant changes in the amplitudes of OP2 wave, dark‐adapted 0.01 b‐wave, 3.0 a‐ and b‐wave, light‐adapted 3.0 a‐ and b‐wave, and light‐adapted 3.0 flicker wave were observed between the operated and fellow eyes at same the time point (Figure 3C‐I).

**FIGURE 4:**
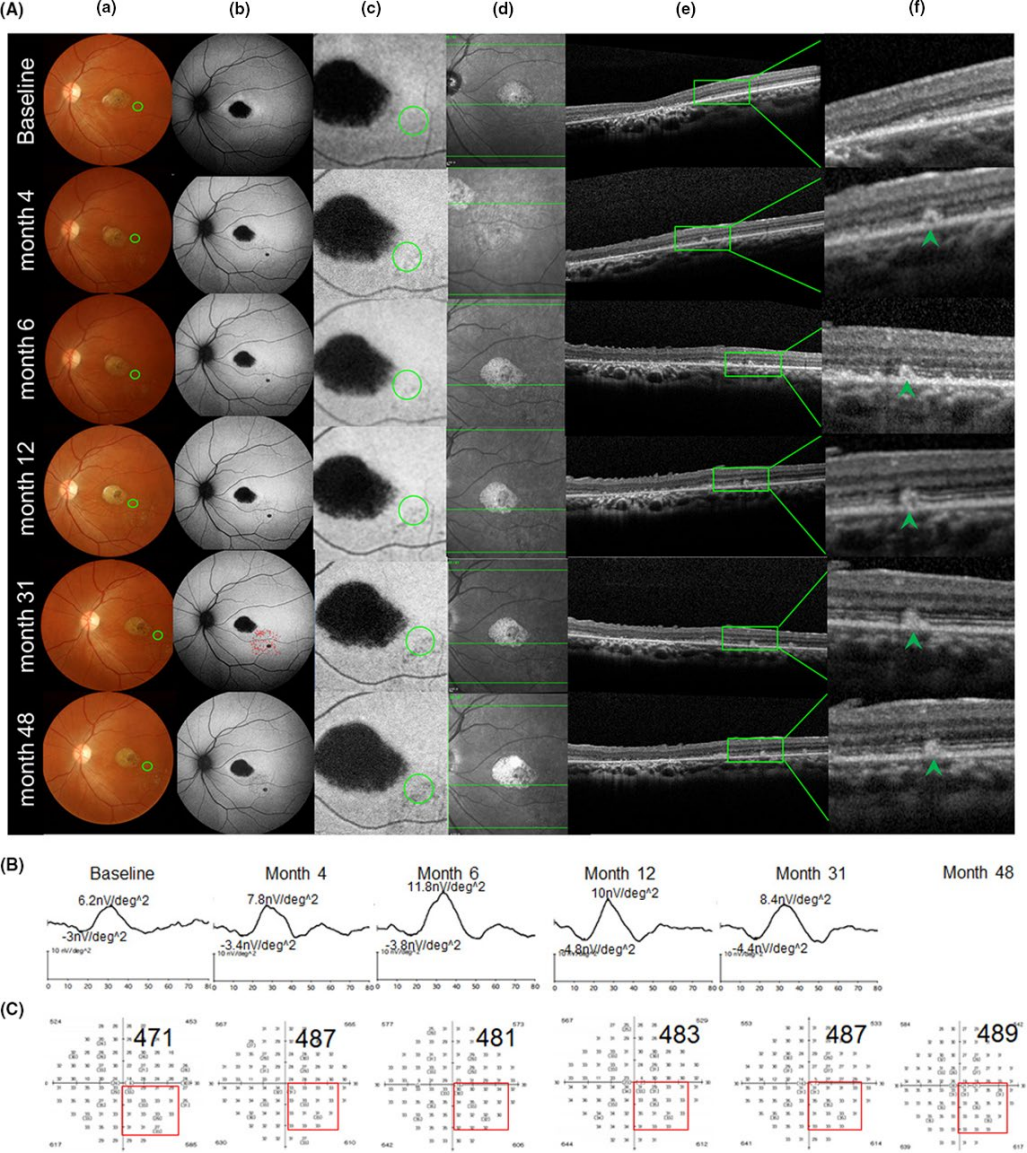
Morphological (A) and function (B,C) change in the operated eye of patient 6, who was classified as a category I patient. No hyperfluorescence dots were observed with the AF inside the first vascular arch of the posterior pole (FVAPP) before operation (baseline b). Fundus photography (a), autofluorescence of the posterior pole (b), typical autofluorescence changed area near macular after surgery (green circle, c), OCT scanning position cross green circle in c (green line in the middle, d), OCT scanning image from green line in d (e), and higher magnification of OCT scanning image from the green box in e, which showed local small high reflection (SHR) (green arrow, f) at each follow‐up time. From baseline to the last visit (48th month), fundus photography (a) and autofluorescence (b) displayed scattered yellowish dots and pigmentation (a) and hypo‐autofluorescence dots (b) around the transplantation site (green circle, a), and OCT scanning (e, f) showed SHR existed (green arrow, f). In the autofluorescence image at the 31st month, we use red dots to label the SHR area (b), which were calculated separately at each time point by multifocal electroretinography (mfERG) with nv/deg^2^ (g) and visual field with decibel value (h)

**FIGURE 5:**
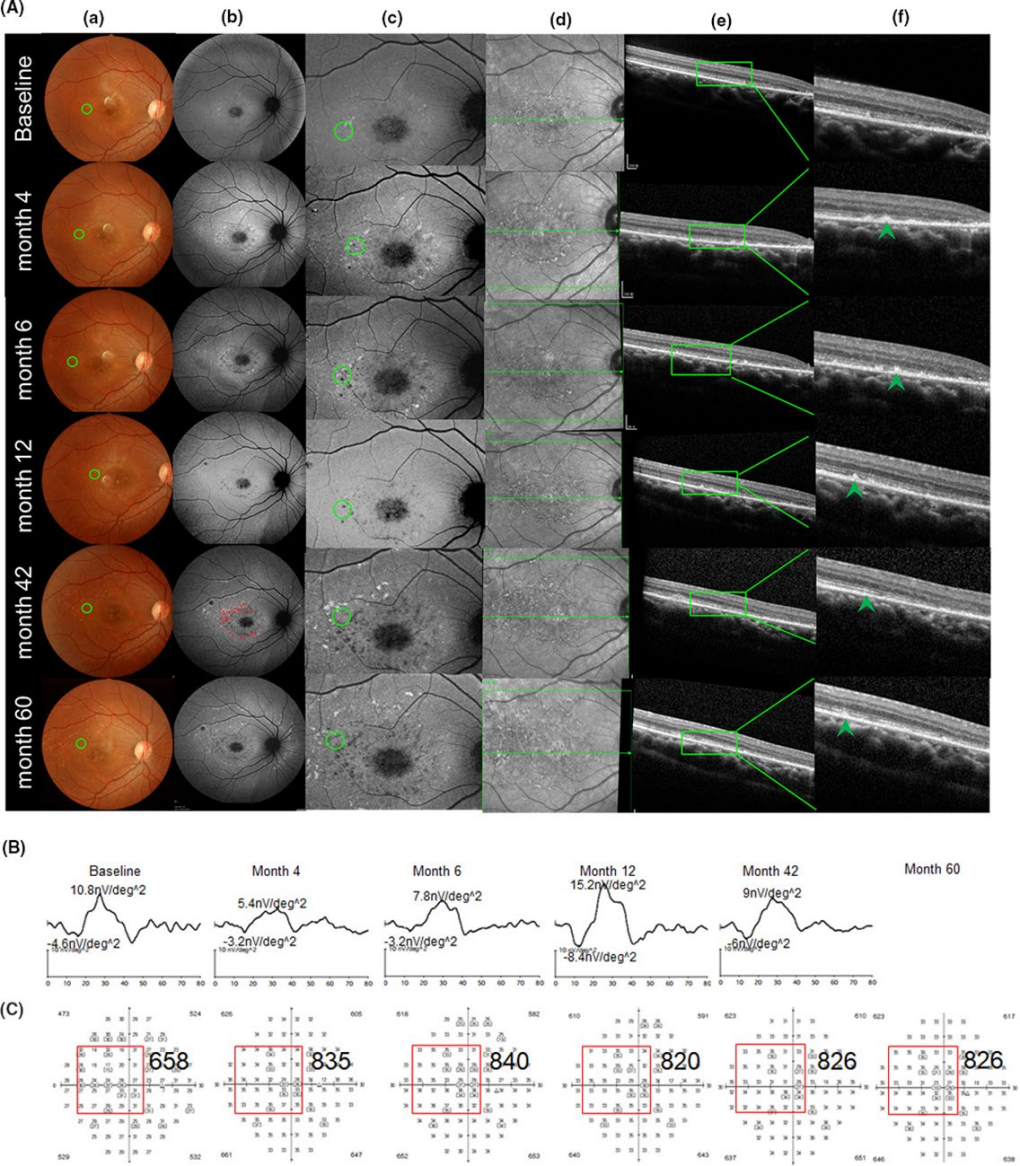
Morphological (A) and function (B,C) change in the operated eye of patient 3, who was classified as a Category II patient. Hyperfluorescence dots were revealed by the AF inside the first vascular arch of the posterior pole (FVAPP) before surgery (baseline b). Fundus photography (a), autofluorescence of the posterior pole (b), typical autofluorescence changed the area near macular after surgery (green circle, c), OCT scanning position cross green circle in c (green line in the middle, d), OCT scanning image from green line in d (e), and higher magnification of OCT scanning image from the green box in e, which showed local small high reflection (SHR) (green arrow, f) at each follow‐up time. From baseline to the last visit (60th month), fundus photography (a) and autofluorescence (b) displayed scattered yellowish dots and pigmentation (a) and hypo‐autofluorescence dots (b) around the transplantation site (green circle, a), OCT scanning (e, f) showed SHR existed (green arrow, f). In the autofluorescence image at the 42nd month, we use red dots to label the SHR area (b), which was calculated separately at each time point by mfERG with nv/deg^2^ (g) and visual field with decibel value (h)

**FIGURE 6:**
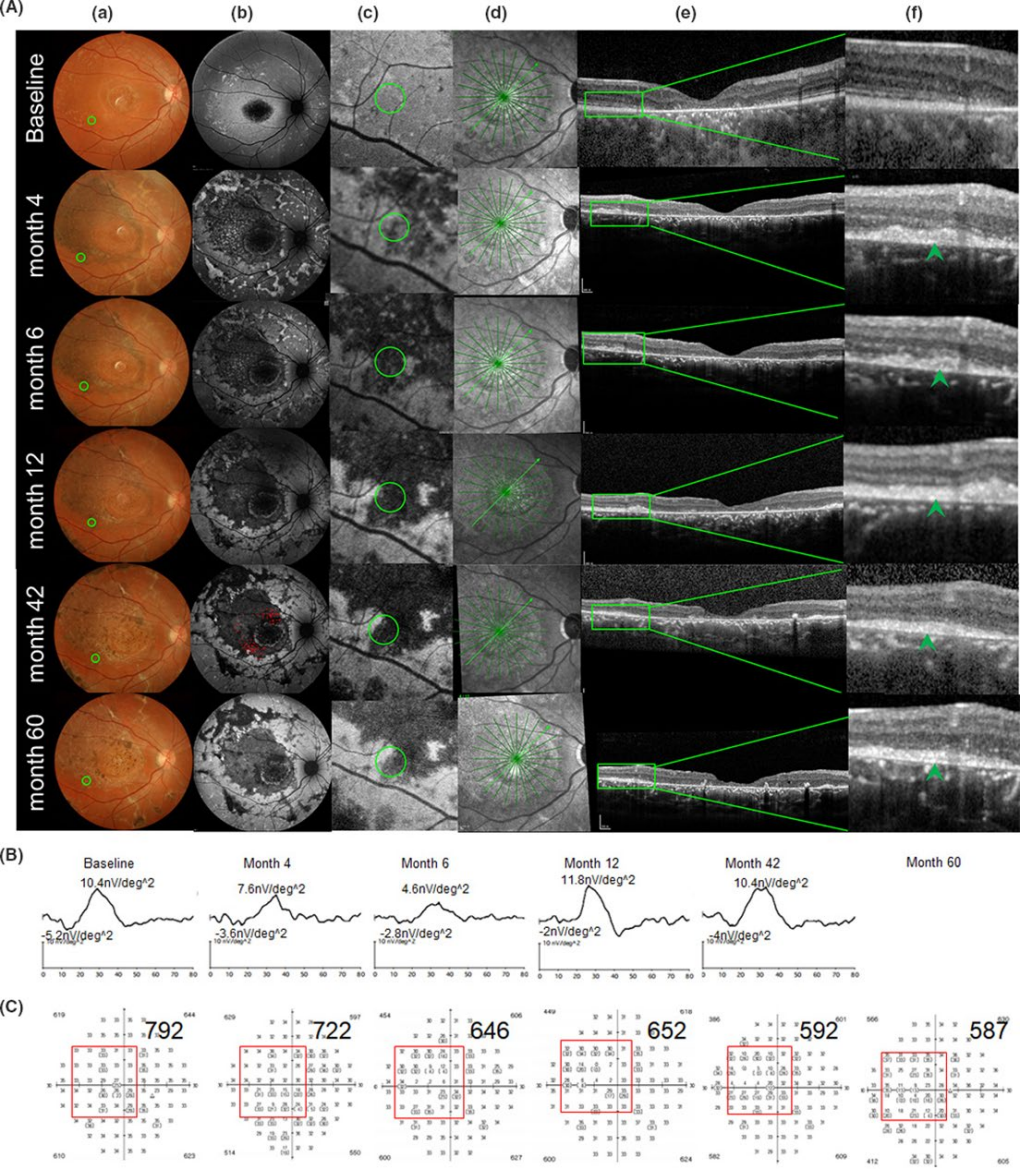
Morphological (A) and function (B,C) change in the operated eye of patient 2, who was classified as a Category III patient. Hyperfluorescence dots were detected by AF inside and outside of the first vascular arch of the posterior pole (FVAPP) before surgery (baseline b). Fundus photography (a), autofluorescence of the posterior pole (b), typical autofluorescence changed the area near macular after surgery (green circle, c), OCT scanning position cross green circle in c (green line in the middle, d), OCT scanning image from green line in d (e), and higher magnification of OCT scanning image from the green box in e, which showed local small high reflection (SHR) (green arrow, f) at each follow‐up time. From baseline to the last visit (60th month), fundus photography (a) and autofluorescence (b) displayed scattered yellowish dots and pigmentation (a) and hypo‐autofluorescence dots (b) around the transplantation site (green circle, a), OCT scanning (e, f) showed SHR existed (green arrow, f). In the autofluorescence image at the 42nd month, we use red dots to label the SHR area (b), which were calculated separately at each time point by mfERG with nv/deg^2^ (g) and visual field with decibel value (h)

**FIGURE 7:**
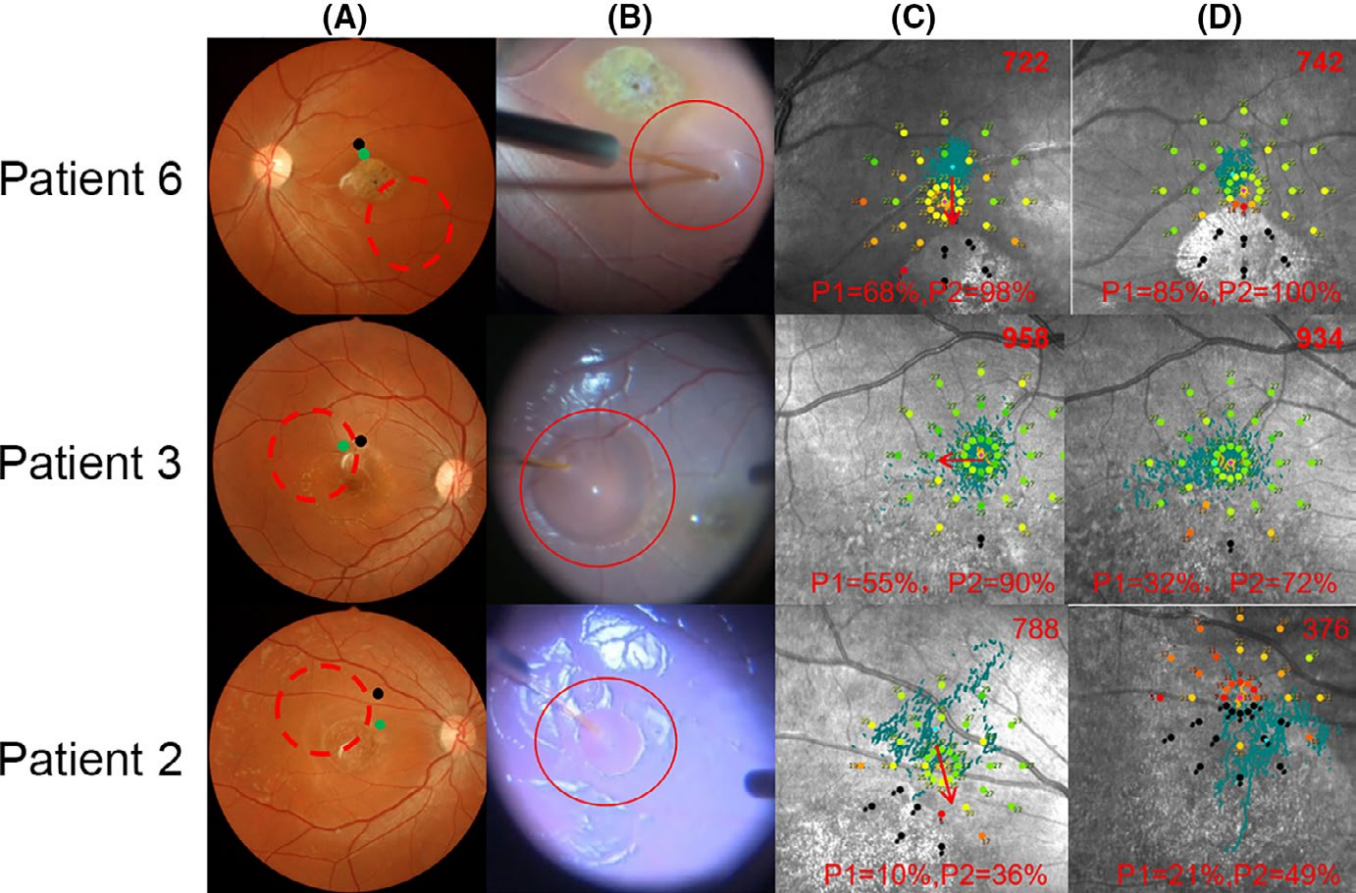
Fundus photography and macular function by microperimetry. Fundus photography before surgery (a): the area of transplanted cells spread (red dash circle), the fixation point shifted from its original position before operation (black dot, a) to a new position 12 months after operation (green dot, a). Fundus photography during the operation (b): Q‐CTS‐hESC‐2‐RPE cells were slowly injected into the subretinal space by a 41‐gauge injection cannula (red circle, b). Microperimetry before (c) and 12 months after surgery (d): the red value on the top right represents the sum of the microperimetry light thresholds, and the value on the bottom means the fixation stability at 1° (P1) and 2° (P2) area. The fixation point was plotted by the blue dots, and shift of fixation point was showed by the red arrow (c)

Based on the pre‐operative fundus autofluorescence (AF), we divided the patients into three categories, as follows: category I: hyperfluorescent dots were completely absent inside the first vascular arch of the pole; category II: hyperfluorescent dots were present within the central retinal area bordered by the vascular arcades; and category III: hyperfluorescent dots extended in areas beyond the retinal vascular arcades. Two cases were classified as category I (P1 and P6), two cases as category II (P3 and P4), and 3 cases as category III (P2, P5 and P7). After the transplantation of hESC‐RPE into the subretinal space of the category I patients, the OCT test detected small high reflective (SHR) foci mainly located above the RPE layer, whereas the AF examination revealed a slight change from hyper‐fluorescence to hypo‐autofluorescence (Figure 4). Four cases with category I, II and III classifications (57%; P6, P3, P4 and P5) displayed an obvious mfERG improvement in the SHR area; two of them (P6 and P3) showed mfERG improvements that were consistent with the improvements in the local light sensitivities as evidenced by visual field test result (Figures 4, 5 and Figure S3, Table S2). The three other patients with category I and III classifications (42%; P1, P2 and P7) displayed a stable mfERG and a stable (P1 and P2) or a slightly decreased (P7) visual field in the SHR area (Figure 6 and Figure S3, Table S2). For the category III patients (P2, P5 and P7), the postoperative AF showed a massive change in hypo‐autofluorescence (Figure 6B,C), whereas the fundus photography showed a pigmentation change (Figure 6A) and OCT displayed only a partial SHR (Figure 6d,e,f) in the AF‐altered area. The long‐term follow‐up revealed that the partial SHR remained stable over time. Interestingly, our microperimetry test revealed that the fixation points in all of the seven patients shifted towards the transplanted area (Figure 7). The fixation stability in microperimetry in all patients were stable or increased slightly (>5%), except in P3 (category II) and P7 (category III) whose fixation stability decreased. Although the category III patients showed a variable degree of light threshold reduction in their previous visual field, they showed a partial improvement in their fixation stability, as seen in P2 and P5 (Figure 7C,D and Table S2). The visual field corresponding to SHR (small high reflective foci) area in OCT showed that Patient 2 and 4 had decreased dB value, while the other patients' visual field dB value remained similar compared to the baseline. Notably, patient 3 had an improved visual field in the SHR area since the 6th post‐operative month and remained similar up to the 60th month, consistent with the improved mfERG in this area. In summary, more patients in the AF category I and II showed slight AF change and improved local retinal function after operation, while more patients in AF category III showed a massive change with hypo‐autofluorescence in AF, pigmentation in fundus photography, and decreased local retinal function after operation.

Considering that the OCT revealed an obvious SHR area in the RPE layer, we examined the RPE function by subjecting both eyes to electrooculography (EOG) during the last follow‐up. The Arden ratio in the EOG of the operated and fellow eyes was within the normal range, and their respective Arden ratios did not significantly differ (Figure S2; n = 4 [P1, P2, P4, and P6]).

## 4 DISCUSSION

Currently, there are no treatments for STGD1, although gene‐ and stem cell‐based therapies are being developed.^18^, ^19^, ^20^ Although the hESC‐RPE cell‐based treatment has improved the vision of advanced stage STGD1 patients during clinical trials, this therapy has evoked serious concerns about the biosafety of hESC‐RPE cells. As such, long‐term studies on early stage STGD1, which is assumed to be a better time window for treatment than the advanced stage STGD1, are warranted.^12^, ^13^, ^17^, ^19^, ^21^, ^22^ In this study, we enrolled seven early stage STGD1 patients and treated them with subretinal transplantation of hESC‐RPE cells. The patients were followed‐up for 5 years for long‐term evaluation of safety and efficacy in visual improvement. Our long‐term study demonstrated that the CTS‐hESC‐RPE cells could be safely implanted into the subretinal space of diseased human eyes by using our established protocol.^8^ Over the 5‐year follow‐up period, none of the patients experienced severe adverse effects, such as abnormal cell proliferation, tumour genesis, immune rejection, retinal detachment, uncontrollable high intraocular pressure, severe bleeding, or other serious ocular or systemic safety issues caused by the transplantation of CTS‐hESC‐RPE cells. One of the most serious concerns in stem cell transplantation is the risk of tumour formation, because a teratoma could grow from a single undifferentiated embryonic stem cell.^23^ In our study, we did not observe any tumour‐like growths after transplantation. Our study suggested that our differentiation protocols^8^, ^13^ produced a sufficiently pure population of differentiated RPE cells so that no tumour had formed even 5 years after transplantation. In agreement with Schwartz's findings,^19^ our results indicated that hESC‐RPE cells can be implanted safely and are well‐tolerated after being seeded in the appropriate site. Moreover, our results indicated that our stem cell differentiation and injection protocols are feasible for hESC‐RPE cell‐based therapy.^24^

Performing unbiased and repeatable assessments of visual function and providing detailed morphological descriptions after intraocular stem cell transplantation have always been challenging. In this study, we used neurophysiological examinations, namely ffERG, mfERG and PVEP, as the objective means of evaluating visual function, besides the use of typical questionnaires and psychophysical examinations for visual acuity and visual fields in the transplanted area. We found that our approaches had the following merits. (1) Our ffERG and mfERG could more reliably identify patients with early‐stage STGD1 because it is more accurate and sensitive than the AF and OCT tests, albeit AF imaging is still widely used as a monitoring tool for disease progression.^1^, ^25^, ^26^ It has been reported that an abnormal fundus appearance is not always consistent with mild functional damage in rod and cone cells in early‐stage STGD1.^1^, ^24^ (2) Our combined visual function assessments enabled monitoring of long‐term efficacy and safety after stem cell transplantation. In our study, no significant differences in visual acuity, PVEP and ffERG were observed between the operated and fellow eyes at the last time point, except in one patient with AF category III. The possible reasons maybe: visual acuity is subjective, PVEP and ffERG reflect the overall function of retina and visual passway, and PVEP also need good fixation of patient's eye for cooperation. However, we found that localized visual function assessment involving mfERG and visual field, together with the measurement of fixation shift in microperimetry, could assess the functional change in the transplanted area. We observed that four cases with category I, II and III classifications had mfERG improvements in the SHR area, and three other cases with category I and III classifications displayed a stable mfERG in the SHR area. (3) We introduced a new AF preoperative subgrouping that could screen the subjects and predict post‐transplantation outcomes in morphology and function in STGD1. Although it was difficult to confirm the fate of transplanted cells in human subjects, the OCT and mfERG provided objective evidence showing changes in retina morphology and visual function. The SHR foci located above the host RPE layer, and which was detected by our OCT, was not a tumour because it did not grow. Rather, it remained stable during the long‐term follow‐up.

The fate of stem cell‐derived RPE cells transplanted in the subretinal space in clinical trial remains unclear. McGill et al reported that the iPS‐derived RPE cells transplanted into the subretinal space of non‐immunosuppressed rhesus monkeys were no longer detectable 3 weeks after transplantation due to rejection by the immune system.^27^ Although the iPS‐derived RPE cells originated from the patient's adult cells and were distinct from the hESC‐RPE cells we applied, it did evoke serious concerns about the intraocular immune response after stem cell transplantation.^19^ Idelson et al^28^ reported that hESC‐RPE cells could inhibit T cell responses in vitro and in vivo. Moreover, Szatmári‐Tóth et al^29^ reported that dying hESC‐RPEs are efficiently engulfed by macrophages, resulting in the release of high amounts of IL‐6 and IL‐8 cytokines. In our studies, all the patients took systemic immunosuppression drugs, as described previously.^8^, ^17^ As such, we did not observe any notable immune responses or inflammation in the treated patients, through the many systemic and local monitoring methods currently available. Instead, we noticed a relatively stable improvement in morphology and local function in AF category I and II patients, as well as a transient morphological restoration in the category III patient, as evidenced by a massive pigmentation in the colour photograph which showed hypo‐autofluorescence by AF.

In agreement with previous findings of Schwartz et al, we observed a deposition of numerous pigments, which appeared to be similar to hypo‐autofluorescence dots, after hESC‐RPE cell transplantation.^19^ There were two possible explanations for the occurrence of hypo‐autofluorescence dots in AF: (1) non‐transparent biological substances covering the RPE layer or (2) some of the original RPE cells had died. In our study, the hypo‐autofluorescence dots in the transplanted area were more likely to have resulted from the existence of non‐transparent biological substances covering the RPE cells, because they looked like black pigmentation in the fundus colour photographs, whereas they appeared as SHR dots in the OCT. In addition, the improved visual functions in the SHR area, such as the improvements in mfERG, the improvements in or the stability of VF, and the fixation shift, could not be explained by significant death among RPE cells. However, the exact mechanism by which SHR dots display hypo‐autofluorescence remains unclear and warrants further investigation. Moreover, we found some hyper‐autofluorescent RPE cells in or near the transplanted area, and this observation may have resulted from the migration of the transplanted cells or from the reaction of the host RPE cells.

In this study, we transplanted an hESC‐RPE suspension into the subretinal space with premade standardized products.^12^, ^13^, ^30^ However, whether the transplanted RPE cells can form a single layer and function as mature RPE cells warrants confirmation.^31^ An alternative therapy for macular tissue regeneration is to use RPE patch grafts. The advantage of this approach is that the graft survives longer and is easily visible, but the large surgical incision required causes mechanical trauma and restricts the patch area.^32^ More recently, we reported subretinal transplantation of c‐Kit^+^/SSEA4^−^‐enriched retinal progenitor cells from hESC‐derived retinal organoids as a promising therapeutic strategy for retinal regeneration.^33^ This phase I clinical trial has demonstrated that hESC‐RPE cell injection into the subretinal space is safe, well‐tolerated and promising in efficacy, and thus warrants further clinical trials for the treatment of properly selected STGD1 patients.

## CONFLICT OF INTEREST

Prof. Qi ZHOU declared that he is the editor‐in‐chief of ‘Cell Proliferation’. The authors do not have other conflicts of interest to declare.

## AUTHOR CONTRIBUTIONS

Shi‐Ying Li, Fang Wang and Liu Wang wrote the manuscript and were involved in the figures, data gathering and literature search. Yong Liu and Shi‐Ying Li were involved in patient surgery, data analysis and interpretation. Zheng‐Qin Yin, Yong Liu, Shi‐Ying Li, Qi Zhou, Liu Wang and Jie Hao were involved in the study design, data analysis, and interpretation. Shi‐Ying Li, Tong‐Tao Zhao and Xiao‐Hong Meng were involved in data analysis and interpretation. Qi‐You Li, Hai‐Wei Xu and Lei Wang were involved in cell preparation and quality control. All authors have seen and approved the final version of the manuscript for publication.

## Supporting information

Figure S1‐3

Table S1‐3

Supplementary Material

Video S1

Video S2

## Data Availability

All supporting data are included in the article and its additional files.
