# Supplementary material for: A phase I clinical trial of human embryonic stem cell‐derived retinal pigment epithelial cells for early‐stage Stargardt macular degeneration: 5‐years' follow‐up
Source: Cell Prolif. 2021 Aug 4;54(9):e13100. doi: 10.1111/cpr.13100 (PMC8450131; doi:10.1111/cpr.13100)
Supplement: Supplementary file 1 — Figure S1‐3 [file CPR-54-e13100-s003.docx]

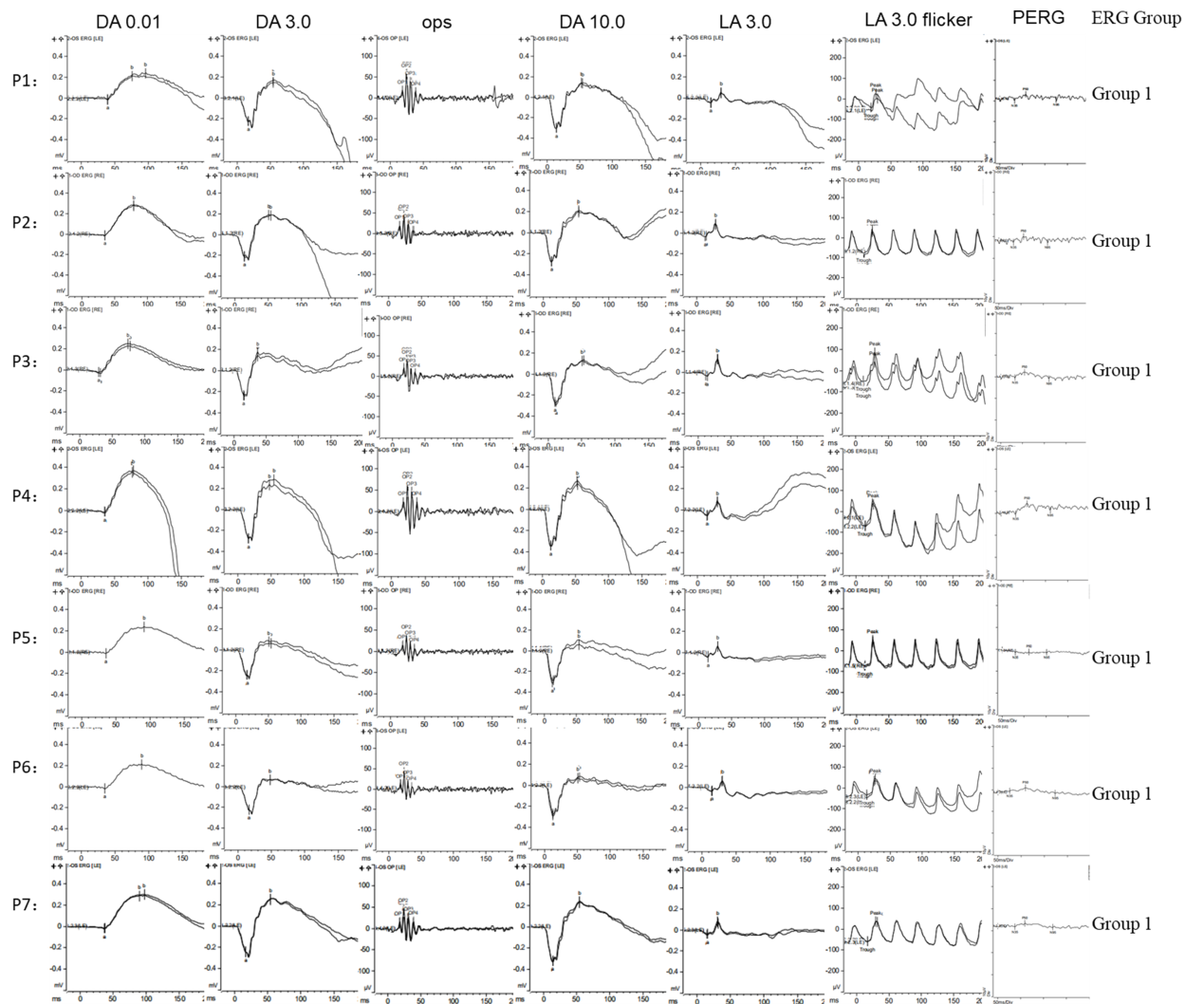
**Figure S1. Waveforms of preoperative ffERG and PERG in all STGD1 patients.** With the use of ffERG Group classification, which is based on waveforms of ffERG and PERG, all patients were classified into as Group 1.



**Figure S2. Arden ratio in the EOG of the operated eyes and the fellow eyes at the last follow-up.** At the last follow-up, both eyes of four patients (P1, P2, P4, and P6) were subjected to an EOG examination. No significant difference in Arden ratio in the EOG was observed between the operated and fellow eyes.


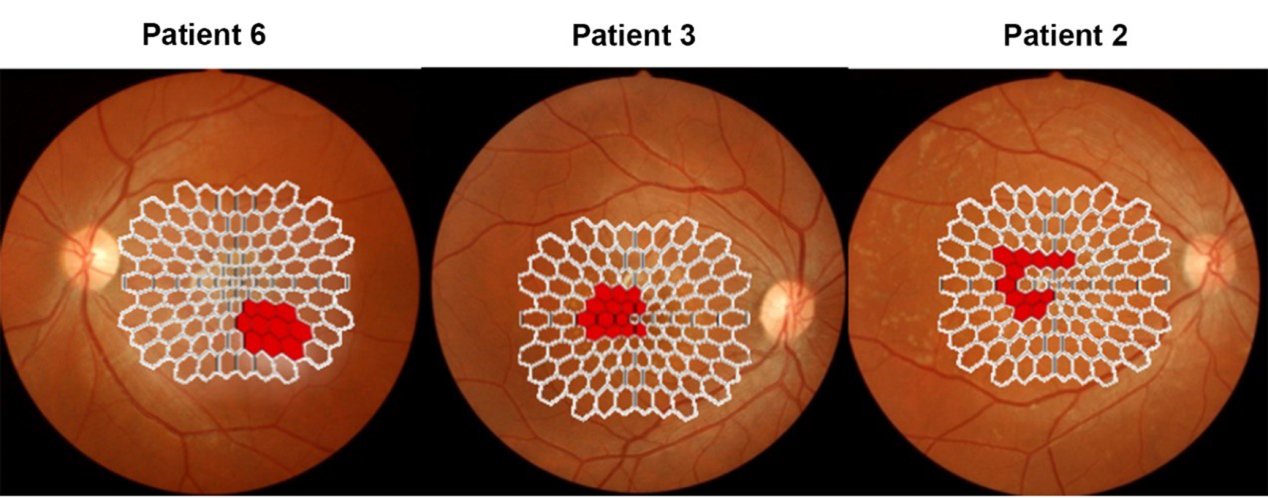
**Figure S3. SHR area were also labeled in the multifocal electroretinography (mfERG) by red hexagon.**
