# Supplementary material for: A phase I clinical trial of human embryonic stem cell‐derived retinal pigment epithelial cells for early‐stage Stargardt macular degeneration: 5‐years' follow‐up
Source: Cell Prolif. 2021 Aug 4;54(9):e13100. doi: 10.1111/cpr.13100 (PMC8450131; doi:10.1111/cpr.13100)
Supplement: Supplementary file 2 — Table S1‐3 [file CPR-54-e13100-s001.docx]

**Table S1. Best-corrected visual acuity (BCVA) in participants**

| NUMBER | ETDRS | Baseline | 1 week after surgery | 1 month after surgery | 3 months after surgery | 6 months after surgery | 9 months after surgery | 12 months after surgery | 42 months after surgery | 60 months after surgery |
| --- | --- | --- | --- | --- | --- | --- | --- | --- | --- | --- |
| P1 | operated eye | 39 | 41 | 41 | 41 | 40 | 41 | 38 | 34 |  |
|  | fellow eye | 39 | 39 | 41 | 41 | 40 | 42 | 39 | 37 |  |
| P2 | operated eye | 40 | 40 | 39 | 40 | 36 | 37 | 37 | 32 | 27 |
|  | fellow eye | 42 | 42 | 43 | 43 | 43 | 43 | 40 | 42 | 38 |
| P3 | operated eye | 40 | 41 | 45 | 43 | 42 | 42 | 41 | 35 | 23 |
|  | fellow eye | 40 | 42 | 43 | 42 | 40 | 43 | 40 | 34 | 24 |
| P4 | operated eye | 44 | 42 | 40 | 42 | 42 | 41 | 42 | 40 | 40 |
|  | fellow eye | 44 | 45 | 47 | 45 | 48 | 47 | 49 | 46 | 40 |
| P5 | operated eye | 20 | 30 | 33 | 25 | 20 | 20 | 20 |  |  |
|  | fellow eye | 47 | 50 | 50 | 48 | 48 | 48 | 47 |  |  |
| P6 | operated eye | 44 | 42 | 47 | 43 | 42 | 42 | 41 | 33* |  |
|  | fellow eye | 44 | 42 | 44 | 43 | 42 | 41 | 33 | 33* |  |
| P7 | operated eye | 23 | 25 | 18 | 21 | 16 | 20 |  |  |  |
|  | fellow eye | 35 | 38 | 38 | 38 | 36 | 35 |  |  |  |

* The last visit for P6 was 48 months after surgery. This patient’s Early Treatment Diabetic Retinopathy Study (ETDRS) letter score decreased by more than 5 letters compared with the baseline, indicating a reduced BCVA; however, the patient’s BCVA remained stable when the comparison was made with EDTRS letter score of the untreated fellow eye.

**Table S2. Participants’ information**

|  | ETDRS | | | The multifocal ERG corresponding to SHR area (nV/deg^2) & | | | The visual field corresponding to SHR area (dB) * | | | The fixation stability of microperimetry (P1, P2) # | | |
| --- | --- | --- | --- | --- | --- | --- | --- | --- | --- | --- | --- | --- |
| Participant number | Baseline | The best | Last time | Baseline | The best | Last time | Baseline | The best | Last time | Baseline | The best | Last time |
| P1 | 39 | 42(1d) | 34(42m) | 8.8 | 12.8(12m) | 10(42m) | 176 | 150(12m) | 122(42m) | P1=69%  P2=98% | P1=78%  P2=100%  (42m) | P1=78%  P2=100%  (42m) |
| P2 | 40 | 40(3m) | 27(60m) | 15.6 | 14.4(42m) | 14.4(42m) | 792 | 722(4m) | 587(60m) | P1=10%  P2=36% | P1=21%  P2=49%  (42m) | P1=21%  P2=49%  (42m) |
| P3 | 40 | 45(1m) | 23(60m) | 15.4 | 23.6(12m) | 15(42m) | 658 | 840(6m) | 826(60m) | P1=55%  P2=90% | P1=57%  P2=94%  (1m) | P1=32%  P2=72%  (60m) |
| P4 | 44 | 42(3m) | 40(60m) | 8.2 | 19.8(42m) | 19.8(42m) | 428 | 418(4m) | 294(60m) | P1=80%  P2=98% | P1=65%  P2=99%  (1m) | P1=20%  P2=54%  (60m) |
| P5 | 20 | 33(1m) | 20(12m) | 7.4 | 11.6(12m) | 11.6(12m) | 1110 | 931(6m) | 894(12m) | P1=5%  P2=17% | P1=28%  P2=75%  (3m) | P1=12%  P2=33%  (12m) |
| P6 | 44 | 47(1m) | 33(48m) | 9.2 | 15.6(6m) | 12.8(36m) | 471 | 489(48m) | 489(48m) | P1=68%  P2=98% | P1=85%  P2=100%  (6m) | P1=85%  P2=100%  (36m) |
| P7 | 23 | 25(2m) | 20(9m) | 11.4 | 13(9m) | 13(9m) | 702 | 571(6m) | 571(6m) | P1=31%  P2=79% | P1=18%  P2=62%  (6m) | P1=7%  P2=48%  (9m) |

Note: SHR (small high reflective foci) in OCT

& mfERG (multifocal ERG): Four cases with category I, II, and III classifications (57%) (Patient 6, Patient 3, Patient 4, and Patient 5) displayed an obvious mfERG improvement in the SHR area; two of them (Patient 6, Patient 3) showed an mfERG improvement that is consistent with the improvement in light sensitivities as evidenced by visual field test result (Figures 4 & 5, Table S2). The three other three patients with category I and III (42%) (Patient 1, Patient 2, and Patient 7) displayed a stable mfERG and a stable (Patient 1, Patient 2) or a slightly decreased (Patient 7) visual field in the SHR area (Figure 6, Table S2).

* The visual field corresponding to SHR (small high reflective foci) area in OCT showed that Patient 2 and 4 had deceased dB value, while the other patients remained visual field value compared to the baseline. Please note Patient3 had improved visual field in the SHR area since the 6^th^ month postoperatively and remained to the 60^th^ month, consistent with the improved mfERG in this area.

#The fixation stability of the microperimetry in all seven patients were stable or increased slightly (> 5%) such as Patient 2, 5 and 6, except in Patient 3 (category II) and Patient 7 (category III) whose fixation stability decreased.

**Table S3 Summary of all adverse events by time of onset**

| **System Organ Classification/ Adverse Event Term** | **SMD**  **N=7** |
| --- | --- |
| **Eye Disorders** |  |
| Anterior Surface Pigment | 0 |
| Blurry Vision | 0 |
| Transient lens opacity | 1 |
| Conjunctival Hemorrhage | 7 |
| Conjunctival Hyperemia | 7 |
| Corneal Abrasion | 0 |
| Dry Eye | 0 |
| Transient high intraocular pressure | 2* |
| Eye Sting Pain | 2 |
| Eye Watery | 0 |
| Floaters | 0 |
| Foreign Body Sensation | 2 |
| Inferior Folds | 0 |
| Posterior Vitreal Detachment | 0 |
| Ptosis | 0 |
| Retinal Vascular Leakage | 0 |
| Suture Granuloma | 0 |
| Cardiovascular Disorders | 0 |
| General Disorders | 0 |
| Gastrointestinal Disorders | 0 |
| Infections | 0 |
| Injury | 0 |
| Investigations | 0 |
| Musculoskeletal Disorders | 0 |
| Neoplasms | 0 |
| Nervous System Disorders | 0 |
| Psychiatric Disorders | 0 |
| Respiratory Disorders | 0 |
| Skin Disorders | 0 |
| Total | 21 |

**Note:** ***** Two out of the seven patients (P4 and P7) had a transient high intraocular pressure ranging from 26 mmHg to 32 mmHg within 1–2 months after the operation, and this pressure build up was relieved by eye drops and was cured after the silicone oil removal at the 3^rd^ month post-transplantation. Other adverse events in the eye were transient and did not require any intervention.
