# Supplementary material for: A phase I clinical trial of human embryonic stem cell‐derived retinal pigment epithelial cells for early‐stage Stargardt macular degeneration: 5‐years' follow‐up
Source: Cell Prolif. 2021 Aug 4;54(9):e13100. doi: 10.1111/cpr.13100 (PMC8450131; doi:10.1111/cpr.13100)
Supplement: Supplementary file 3 — Supplementary Material [file CPR-54-e13100-s002.docx]

**Inclusion and exclusion criteria**

Patients who met the following criteria were recruited: 1) aged 18–65 years and signed an informed consent; 2) disease-causing variants were confirmed by eye gene-enriched panel based next-generation sequencing (NGS) or whole exome sequencing (WES), in silico molecular genetic analysis and co-segregation analysis. Finally, the pathogenicity class of these variants was confirmed according to the American College of Medical Genetics (ACMG) guidelines. 3) visual impairment due to STGD1 in both eyes; 4) BVCA score of 19–73 letters (approximately equivalent to Snellen’s 20/400 to 20/40) when the testing distance is 4 meters, with the ETDRS as the visual acuity checklist; 5) able to adhere to the scheduled follow-up visits and to the protocol-specified treatment period; 6) full field electroretinography result indicating macular dysfunction only, without generalized rod and cone dysfunction.

The exclusion criteria were as follows: 1) eyes with active intraocular inflammation, infection, or concomitant diseases, including severe cataract, glaucoma, retinal vascular obstruction, retinal detachment, macular hole, vitreous macular traction, and choroidal neovascularization; 2) a history intraocular surgery; 3) a history of stroke, coronary heart disease, renal insufficiency or kidney transplantation, and other systemic chronic diseases; 4) anaphylactic reaction to sodium fluorescein; 5)hypertension (systolic pressure of >140 mmHg or diastolic pressure of >90 mmHg) or diabetes that could not be controlled by medication; 6) females who intended to get pregnant within the next 6 months, as well as those who were pregnant or lactating.

**Detailed Clinical evaluation Methods**

The blood test for safety included routine blood test, liver and kidney function, interleukin-6 (IL-6), procalcitonin (PCT), multi-tumor markers, and myocardial enzyme tests. The routine blood and liver and kidney function tests were performed at baseline and at 1, 2, 3, 6, 9, 12, 24, 36, 42 and 60 month after operation. The blood tests of IL-6, PCT, multi-tumor markers, and myocardial enzyme were done at baseline and at 1, 6, 12, 24, 36, 42 and 60 month after operation. The multi-tumor markers are consisted of [alpha](https://cn.bing.com/dict/search?q=alpha&FORM=BDVSP6&mkt=zh-cn)-[fetoprotein](https://cn.bing.com/dict/search?q=fetoprotein&FORM=BDVSP6&mkt=zh-cn), [carcinomacmbryonic](https://cn.bing.com/dict/search?q=carcinomacmbryonic&FORM=BDVSP6&mkt=zh-cn) [antigen](https://cn.bing.com/dict/search?q=antigen&FORM=BDVSP6&mkt=zh-cn), cytokeratin 19 fragment, neuron specific enolase, and carbohydrate antigens 125 and 242.

Slit-lamp biomicroscope and tonometer were performed by HAAG-STREIT (Switzerland). Retina morphology was monitored by fundus photography (Kowa, nonmyd, Japan), fundus autofluorescence and fundus fluorescence angiography (HRA2, Heidelberg, Germany), *S*pectral domain optical coherence tomography (HRA2, Heidelberg, Germany). Visual function was evaluated by the best-corrected visual acuity with ETDRS chart (230-E-P, Precision Vision, USA), visual field tests and electrophysiology. The visual field tests included perimeter in 30 degree (Humphrey Field Analyzer II, Germany) and microperimetry in 10 degree (Maia MP1, Italy). The visual electrophysiology included full-field electroretinography and pattern visual evoked potential (Espion system, Diagnosys LLC, Lowell, MA, USA) , and mutifocal electroretinography (mfERG). To monitor the local retinal function objectively, the mfERG testing was performed using a 103-element scaled stimulus array, and incorporated a real-time fundus camera, allowing fixation-dependent stimulus placement to be monitored during the examination (Veris system, ElectroDiagnostic Imaging, Inc., Burlingame, CA, USA; CCD Camera, Hitachi Kokusai Electric Inc, Japan). All visual electrophysiology tests were performed according to the International Society for Clinical Electrophysiology of Vision (ISCEV) Standards.
